# Deciphering the *in vitro* mucin-driven interaction dynamics of a synthetic gut bacterial community

**DOI:** 10.1128/msphere.00289-26

**Published:** 2026-07-10

**Authors:** Chong Yu, Jiayi Ao, Mengran Long, Ziqiong Xu, Shaohui You, Yanlei Jiao, Songling Zhu, Shu-Lin Liu, Shuang Wang, Hongxia Bao

**Affiliations:** 1 Genomics Research Center, Key Laboratory of Gut Microbiota and Pharmacogenomics of Heilongjiang Province, State-Province Key Laboratory of Biomedicine-Pharmaceutics of China, College of Pharmacy, Harbin Medical University 34707, Harbin, China; 2 Department of Biopharmaceutical Sciences (State-Province Key Laboratories of Biomedicine-Pharmaceutics of China), College of Pharmacy, Harbin Medical University 34707, Harbin, China; University of Michigan Medical School, Ann Arbor, Michigan, USA

**Keywords:** gut microbiota, mucin, synthetic microbiome, microbial interactions

## Abstract

The gut microbiota is a complex microbial community that plays a crucial role in host health. Environmental and biological factors within the ecosystem influence the dynamic interactions among its members. Although dietary and host-derived nutrient availability play a key role in shaping microbial ecology and interaction patterns, the dynamics of these interactions within the mucus layer remain poorly understood. In this study, we analyzed a synthetic community comprised of six species with variable abilities to utilize mucin. We performed *in vitro* growth analyses and monitored the interactions among community members in monoculture, co-culture, and community batch culture under different nutrient conditions. Our results showed that positive interactions were prevalent among bacteria when mucin served as the sole carbon source. In contrast, the addition of glucose or high nutrient availability significantly increased inter-bacterial competition. These findings suggest that mucin mitigates competitive antagonism and potentially promotes community diversity. Further *in vivo* studies supported the role of mucin in increasing community diversity and modulating bacterial metabolic patterns. Deciphering these intricate relationships is essential for understanding how gut microbiota stability is maintained, and what factors might disrupt this delicate balance.

**IMPORTANCE:**

The gut microbiota is essential for host health, yet microbial interactions within the intestinal mucus layer remain poorly understood. Current understanding of gut microbial ecology is largely based on nutrient-rich media that do not accurately reflect the mucosal environment. Here, we demonstrate that when bacteria rely solely on mucin as a carbon source, cooperative interactions predominate. In contrast, the introduction of simple sugars shifts the balance toward intensified interbacterial competition. Mucin mitigates competitive antagonism, promotes resource utilization, and enhances community diversity. By demonstrating that mucus actively shapes microbial interaction patterns, this study provides a mechanistic framework for understanding gut ecosystem resilience. Furthermore, these findings support the development of more physiologically relevant *in vitro* models for predicting gut microbial dynamics and may guide microbiome-based therapies.

## INTRODUCTION

The human gastrointestinal tract harbors a dense and diverse collection of microorganisms, known as the gut microbiota, which play a vital role in human health (1). The gut microbiota is composed primarily of five bacterial phyla: Bacteroidota (Bacteroidetes), Bacillota (Firmicutes), Actinomycetota (Actinobacteria), Verrucomicrobiota, and Pseudomonadota (Proteobacteria) phylum (2, 3). The healthy adult intestinal microbiota typically comprises more than 150 bacterial species, most of which belong to these phyla (2, 4). Microbial density along the gastrointestinal tract exhibits a distinct gradient, progressively increasing from the stomach to the rectum, with the large intestine harboring the highest density of microorganisms (10^11^ cells/g feces) (5, 6).

Nutrient availability is a crucial factor influencing the composition and function of the gut microbiota (7). Complex carbohydrates, specifically glycans derived from dietary fiber and host-derived mucins, serve as major energy sources for many gut bacteria (8). Mucins, the principal components of mucus, are large and complex glycoproteins synthesized and secreted by goblet cells within the intestinal epithelium (9). In the large intestine, the mucus layer consists of a dense inner layer virtually free of bacteria, and a loose outer layer colonized by abundant gut microbes (10, 11). Microorganisms residing within the mucus layers are called mucosa-associated bacteria (9, 12, 13). It has also been reported that various bacterial taxa are enriched in the mucus layer, including members of the genera *Bacteroides*, *Bifidobacterium*, *Ruminococcus*, and *Clostridium*; the family *Enterobacteriaceae*; and specific species such as *Akkermansia muciniphila* and *Faecalibacterium prausnitzii* (9, 14). Some of these symbionts are mucus-degrading bacteria, such as *A. muciniphila*, while some are not, for example, *Escherichia coli*. This observation suggests the existence of competition or cross-feeding interactions among bacteria at the colonic mucus layer.

Maintaining the integrity of the mucus layer is essential for host health. Previous studies have implicated that reduced or abnormal mucus production may lead to intestinal inflammation (9, 14). When dietary nutrients are abundant in the gut, mucin degradation by microbes decreases. However, when nutrient availability declines, gut microbes utilize mucin as an alternative energy source (15). This shift in resource utilization is likely to alter interaction networks within the gut ecosystem. The complex interplay of relationships, from competition to cooperation between community members, will shape the composition, stability, and niche availability within microbial ecosystems (16).

In this study, we aimed to investigate how nutrient availability influences microbial interactions within the intestinal mucus environment. We assembled a synthetic community consisting of six mucosa-associated bacteria representing the major phyla of the human gut microbiota and investigated their interaction patterns under different nutrient conditions. We found more competitive interactions between species under nutrient-rich conditions compared with the low-nutrient condition. Supplementation with mucin glycans reduced negative interactions and increased positive interactions, resulting in enhanced community diversity both *in vitro* and *in vivo*. This study sheds light on the intricate dynamics of microbial interactions within the gut, particularly the influence of nutrient availability and mucin utilization on community structure and stability.

## RESULTS

### Design of the human minimal microbiome

**Fig 1:**
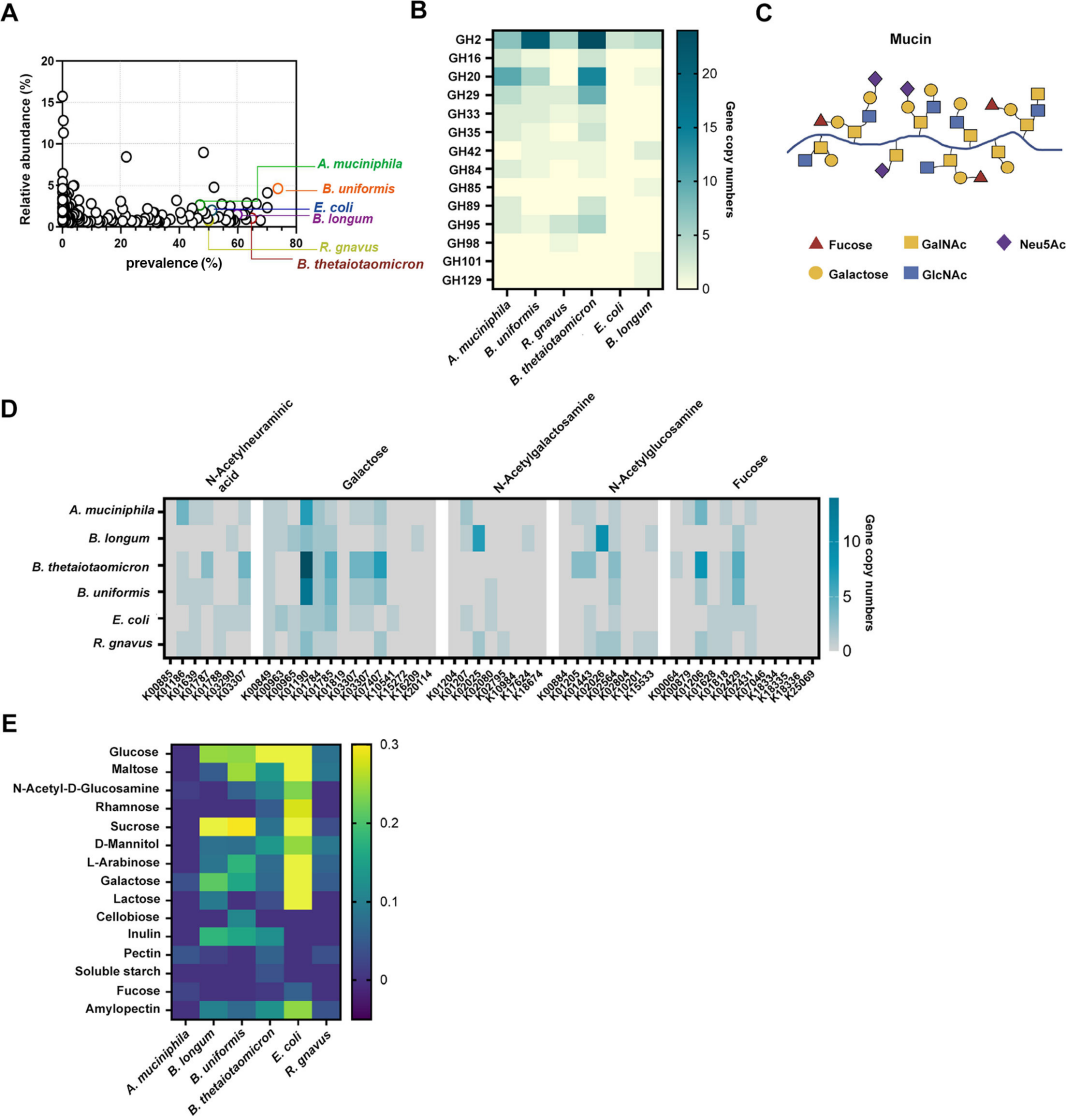
Design of the mucus-associated human minimal microbiome. (**A**) The minimal microbial community was selected based on its prevalence (>25%) and abundance (>0.5%) in the human colonic microbiota, yielding 56 core species (number of samples: *n* = 16,282). (**B**) Mucin-associated glycosyl hydrolases (GHs) encoding heatmap of bacteria based on genomic information. (**C**) Schematic diagram of mucin glycans. GalNAc, N-acetyl galactosamine; GlcNAc, N-acetyl glucosamine; Neu5Ac, sialic acid. (**D**) Genomic potential based on gut metabolic modules for mucin metabolite degradation of selected strains. The scale on the right of the panel represents the gene copy number of genes involved in glycan degradation, as annotated by the KEGG Orthology (KO) database. Darker colors correspond to higher copy numbers, while lighter or absent colors indicate lower copy numbers or absence of the gene. (**E**) Heatmap showing the carbon source utilization potential (OD24h-OD0h) of each synthetic community member in DMM medium. The black boxes indicate no growth.

To model nutrient utilization dynamics within the gut microbiota, we designed a minimal microbial community based on prevalence (>25%) and abundance (>0.5%) of bacterial species in the human colonic microbiota. We first analyzed the health-associated species from the GMrepo v2 database (17) and obtained 56 core species (Table S1). Then we chose six representative strains from the phyla Bacteroidota, Bacillota, Actinomycetota, Verrucomicrobiota, and Pseudomonadota, which have been reported as potential mucin degraders or abundant colonizers of the human gut mucus layer (9, 18, 19). The resulting minimal microbial community consisted of *Bacteroides thetaiotaomicron*, *Bacteroides uniformis*, *Bifidobacterium longum*, *Ruminococcus gnavus*, *Akkermansia muciniphila,* and *Escherichia coli* (Fig. 1A; Fig. S1). Among these strains, *B. uniformis*, *B. longum*, *A. muciniphila*, and *E. coli* were isolated from human stool samples in this study (Table S2). Genomic analysis revealed that *B. thetaiotaomicron*, *B. uniformis*, and *A. muciniphila* encode a relatively high abundance of glycoside hydrolases (GHs) involved in mucin glycan utilization (20), suggesting an enhanced capacity for mucin degradation in these strains (Fig. 1B). However, no interspecies differences were observed in the predicted metabolic pathways for mucin O-glycan core units utilization (Fig. 1C and D). All six species showed limited predicted metabolic activity toward N-acetylgalactosamine but exhibited higher metabolic activity toward galactose (Fig. 1D). We then evaluated the *in vitro* growth of the six bacteria on a panel of 17 mono- and polysaccharides (Fig. 1E). The two *Bacteroides* species, together with *B. longum*, showed broader utilization of plant-derived polysaccharides. In contrast, *E. coli* primarily utilized simple sugars, whereas *R. gnavus* and *A. muciniphila* displayed a preference for host-derived glycans. These results reveal clear metabolic niche differentiation among the six gut bacterial species.

### Monoculture growth behavior of individual strains

**Fig 2:**
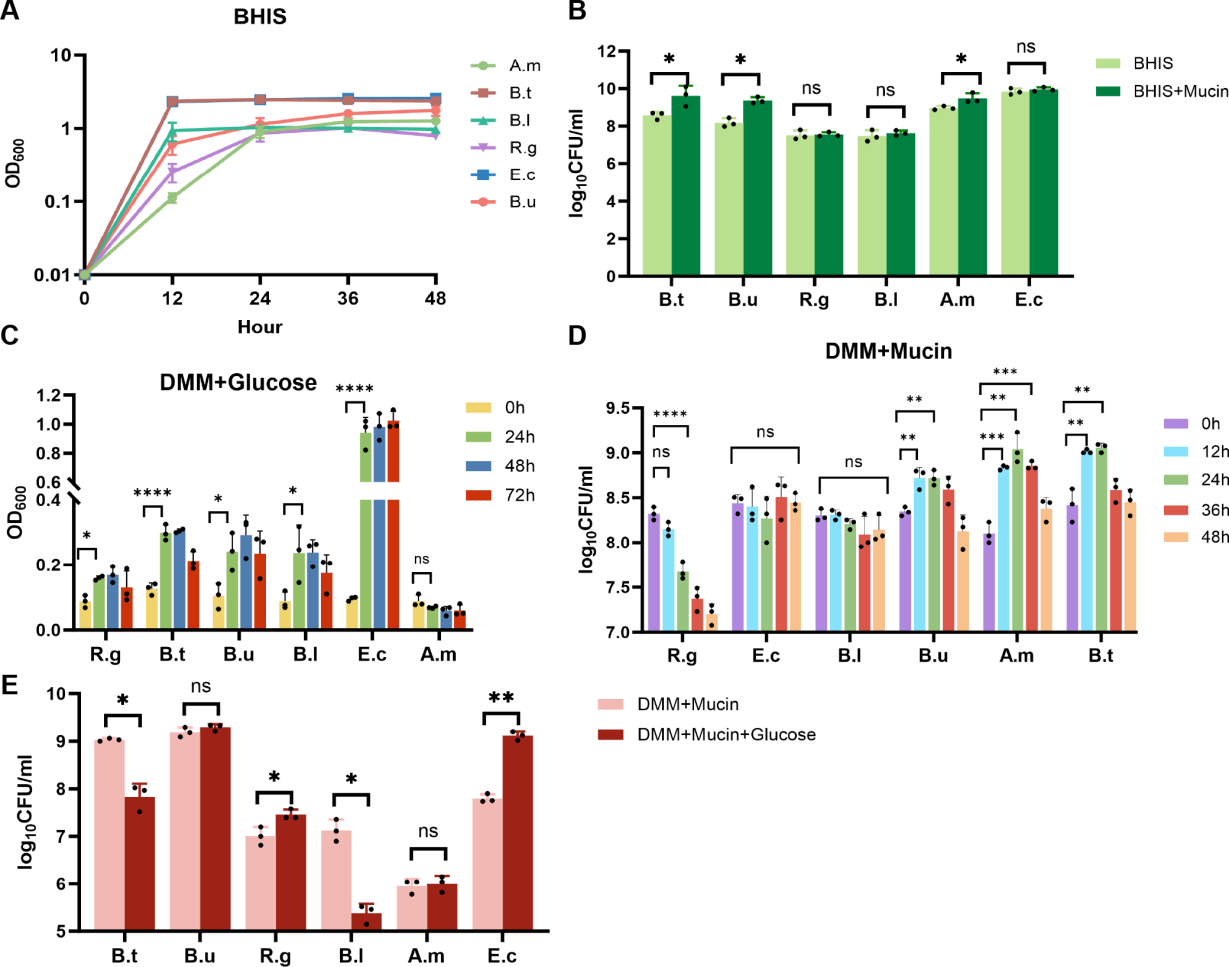
Monoculture growth behavior of individual strains. (**A**) Growth curve of six bacterial species in BHIS medium. Bacteria were cultured in BHIS for 48 h. Data are plotted on a semi-logarithmic scale. (**B**) Effect of mucin on bacterial growth in BHIS medium. Bacteria were cultured for 48 h in BHIS broth with and without 0.5% mucin. Values are shown as mean ± SD from at least three independent experiments (*n* ≥ 3). Statistical significance was determined by Student’s *t*-test. *P < 0.05; ns, not significant. (**C**) The growth of bacterial strains on DMM medium with 0.5% glucose. OD_600_ was determined at 0, 24, 48, and 72 h time points. Data are mean ± SD (*n* = 3). Statistical analysis was performed using one-way ANOVA. *P < 0.05, ****P < 0.0001; ns, not significant. (**D**) The bacterial growth in DMM medium with 0.5% mucin as the sole carbon source. CFU was assessed at 12-h intervals. Data are mean ± SD (*n* = 3). One-way ANOVA, **P < 0.01, ***P < 0.001, ****P < 0.0001; ns, not significant. (**E**) Effect of glucose on bacterial growth in DMM with 0.5% mucin. Data are presented as mean ± SD from at least three independent experiments. Significance was assessed by Student’s *t*-test (*P < 0.05, **P < 0.01; ns, not significant).

We assessed the growth of the six strains in nutrient-rich brain heart infusion-supplemented medium (BHIS) and a defined M9 minimal medium (DMM) (Table S3). As shown in Fig. 2A, BHIS supported the growth of all six strains (Fig. 2A). Supplementation of BHIS with 0.5% porcine gastric mucin significantly increased the colony-forming units (CFUs) of *A. muciniphila*, *B. uniformis*, and *B. thetaiotaomicron* (Fig. 2B), whereas no significant effects were observed for the other three strains (Fig. 2B). In DMM with glucose, *R. gnavus*, *B. thetaiotaomicron*, *B. longum*, *B. uniformis*, and *E. coli* exhibited distinct growth yield and kinetics (Fig. 2C). When 0.5% mucin was provided as the sole carbon source, growth was observed for *B. thetaiotaomicron*, *B. uniformis*, and *A. muciniphila* (Fig. 2D). Although *B. longum* (*P* = 0.3706) and *E. coli* (*P* > 0.9999) did not exhibit significant growth*,* both maintained stable CFU counts over 48 h (Fig. 2D). Interestingly, *R. gnavus*, which has been reported as a mucin-degrader (21), showed a consistent decline in CFU counts under the same conditions (Fig. 2D). To further evaluate the effect of nutrient supplementation, we added 0.25% glucose to DMM containing 0.5% mucin and assessed the CFU after 48 h of culture. Glucose supplementation triggered the growth of *R. gnavus* and *E. coli* compared with the glucose-free control, while *A. muciniphila* and *B. uniformis* were not significantly affected (Fig. 2E). Unexpectedly, the CFUs of *B. longum* and *B. thetaiotaomicron* were significantly decreased following glucose addition (Fig. 2E). Further analysis showed that glucose supplementation initially increased the growth of *B. longum* and *B. thetaiotaomicron* at 24 h, yet led to a significant decrease at 48 h (Fig. S2A and B), suggesting nutrient supplementation can alter bacterial growth dynamics. We further demonstrated that bacterial growth in DMM supplemented with mucin was supported primarily by mucin O-glycans (Fig. S2C through E).

### Bacterial interaction patterns under low-nutrient conditions

**Fig 3:**
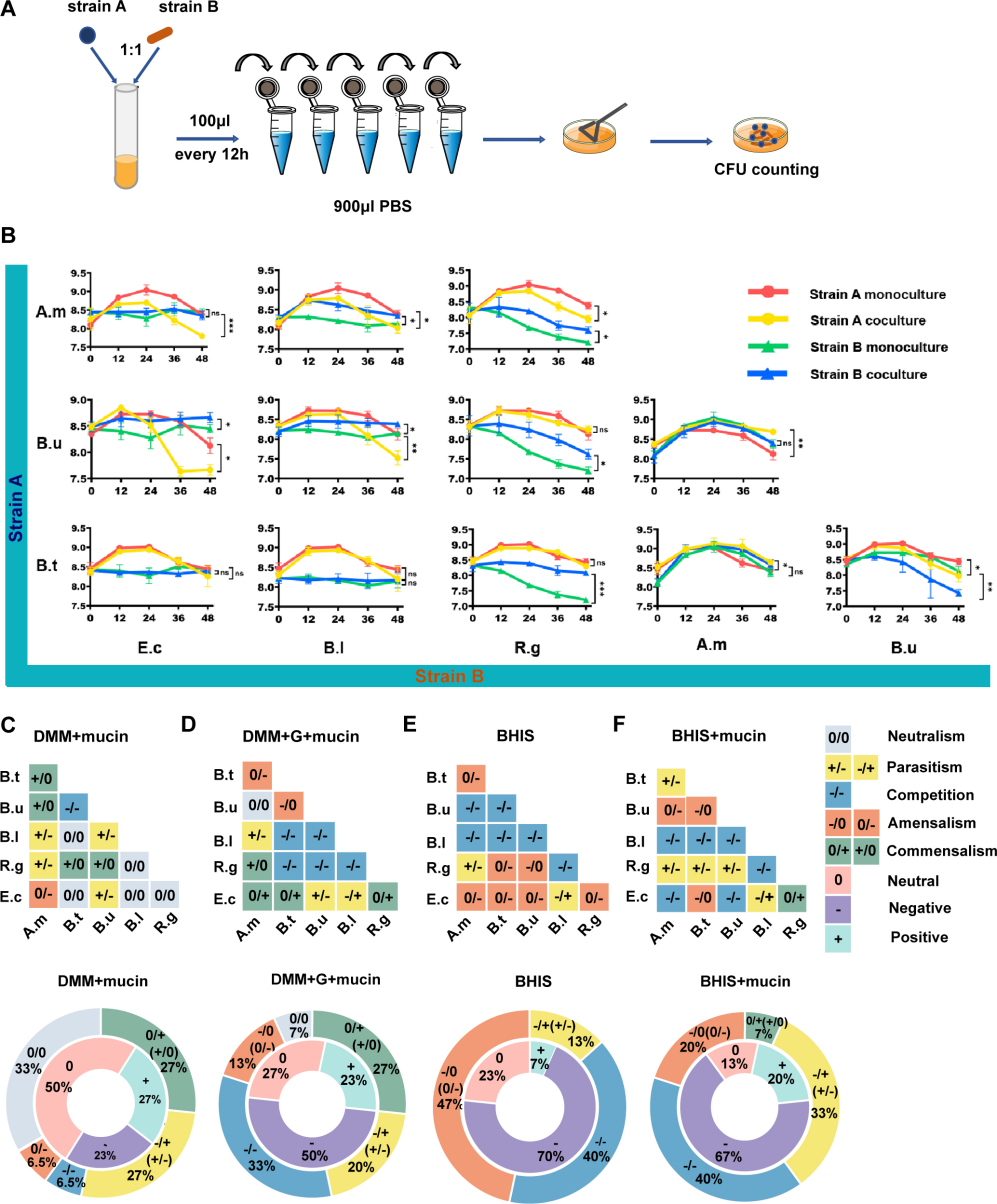
Bacterial interactions in pairwise co-culture experiment. (**A**) Flowchart depicting co-culture experimental design. (**B**) Time-dependent growth dynamics of bacterial species (**A and B**) grown individually (monoculture) and together (co-culture) in DMM with mucin. The growth, measured as log_10_ CFU/mL over time, is shown as mean values (*n* = 3) with standard deviation error bars. The lines represent: Species A monoculture (red), Species B monoculture (green), Species A in co-culture (yellow), and Species B in co-culture (blue). Statistical significance indicated by asterisks corresponds to endpoint analyses shown in panel C (unpaired two-tailed Student’s *t*-test; *P < 0.05, **P < 0.01, ***P < 0.001, and ****P < 0.0001). (**C–F**) Pairwise interaction triangular matrix and statistical graphs of interaction patterns in different nutrient conditions. The abundance ratio (R) of each strain was calculated by dividing the CFU in co-culture by the CFU in monoculture at the 48-h time point. If the CFU ratio increased (*r* > 1) or decreased (*r* < 1) significantly (*P* < 0.05), the bacteria interaction mode was categorized as positive (+) or negative (−). Otherwise, the interaction mode was categorized as neutral (0, *r* = 1). Interaction pattern of bacterial pairs was determined in (**C**) DMM supplemented with 0.5% mucin, (**D**) DMM supplemented with 0.5% mucin plus 0.2% glucose, (**E**) BHIS medium alone, and (**F**) BHIS supplemented with 0.5% mucin.

As demonstrated above, *B. thetaiotaomicron*, *B. uniformis*, and *A. muciniphila* could utilize mucin glycans as the sole carbon source. Therefore, each of the three strains was co-cultured with the other five bacterial strains in DMM supplemented with 0.5% mucin as the sole carbon source at a 1:1 inoculation ratio (OD_600_) for 48 h. Bacterial CFUs were monitored every 12 h, distinguished by antibiotic susceptibility or distinct colony morphology (Fig. 3A; Fig. S3, Tables S4 and S5). As shown in Fig. 3B, *A. muciniphila* significantly promoted the growth of four of the five strains, with the exception of *E. coli. B. thetaiotaomicron*, *E. coli*, *B. longum*, and *R. gnavus* significantly inhibited the growth of *A. muciniphila* in co-culture on mucin. *B. thetaiotaomicron* and *B. uniformis*, members of the same genus, inhibited each other’s growth (Fig. 3B). Additionally, *B. uniformis* promoted the growth of *E. coli*, *B. longum,* and *R. gnavus,* while its own growth was inhibited by *E. coli* and *B. longum*. However, *B. thetaiotaomicron* promoted only *R. gnavus* and was not inhibited by any of the other strains, suggesting marked differences in their interaction patterns (Fig. 3B). Although *B. longum* itself cannot utilize mucin, it inhibited *B. uniformis* and *A. muciniphila*, both of which promoted the growth of *B. longum* (Fig. 3B). As shown in Fig. 3C, when mucin served as the sole carbon source, bacterial interactions were dominated by neutralism (33%), commensalism (27%), and parasitism (27%). In contrast, amensalism (7%) and competition (7%) occurred relatively infrequently, and no mutualistic (+/+) interactions were observed. To investigate how nutrient availability influences bacterial interactions, we studied bacteria’s pairwise interactions in DMM medium with 0.5% mucin plus 0.25% glucose. After adding 0.25% of glucose, the interaction mode was significantly altered (Fig. 3D). We observed a decrease in amensalism (13%) and neutralism (7%), but a significant increase in competition (33%). This result suggests that adding glucose as an alternative nutrient source promoted negative interactions between the community members (Fig. 3D).

### Bacterial interaction is altered by mucin in nutrient-rich media

To further investigate bacterial interactions in a nutrient-rich environment, we co-cultured bacterial pairs in BHIS medium for 48 h and compared the CFUs with those obtained from monocultures. The co-culture interaction matrix revealed widespread negative interactions, including competition (40%), amensalism (47%), and parasitism (13%) (Fig. 3E). The positive interactions were limited to *A. muciniphila-*mediated promotion of *R. gnavus* growth and *E. coli*-mediated promotion of *B. longum* growth, both of which were also observed in DMM supplemented with mucin and glucose. In contrast to the nutrient-limited condition, commensalism was entirely absent in BHIS medium, whereas a substantial number of amensalistic interactions emerged (Fig. 3E). These results further suggest that a nutrient-rich environment may amplify negative interactions and reduce positive interactions between these bacterial strains.

We then investigated bacterial interactions in BHIS supplemented with 0.5% mucin. Although competition remained the dominant interaction type, mucin supplementation led to an increase in commensalism (7%) and parasitism (33%), accompanied by a decrease in amensalism (20%) (Fig. 3F). Comparison of the proportions of positive, negative, and neutral interactions revealed a significant increase in positive interaction but a decrease in neutral and negative interactions (Fig. 3F). These results indicate that mucin enhances the opportunities for positive interactions among bacteria in a nutrient-rich environment. By mitigating negative interactions, mucin may promote bacterial diversity under nutrient-rich conditions.

### Spent culture medium-based bacterial interaction in nutrient-rich condition

**Fig 4:**
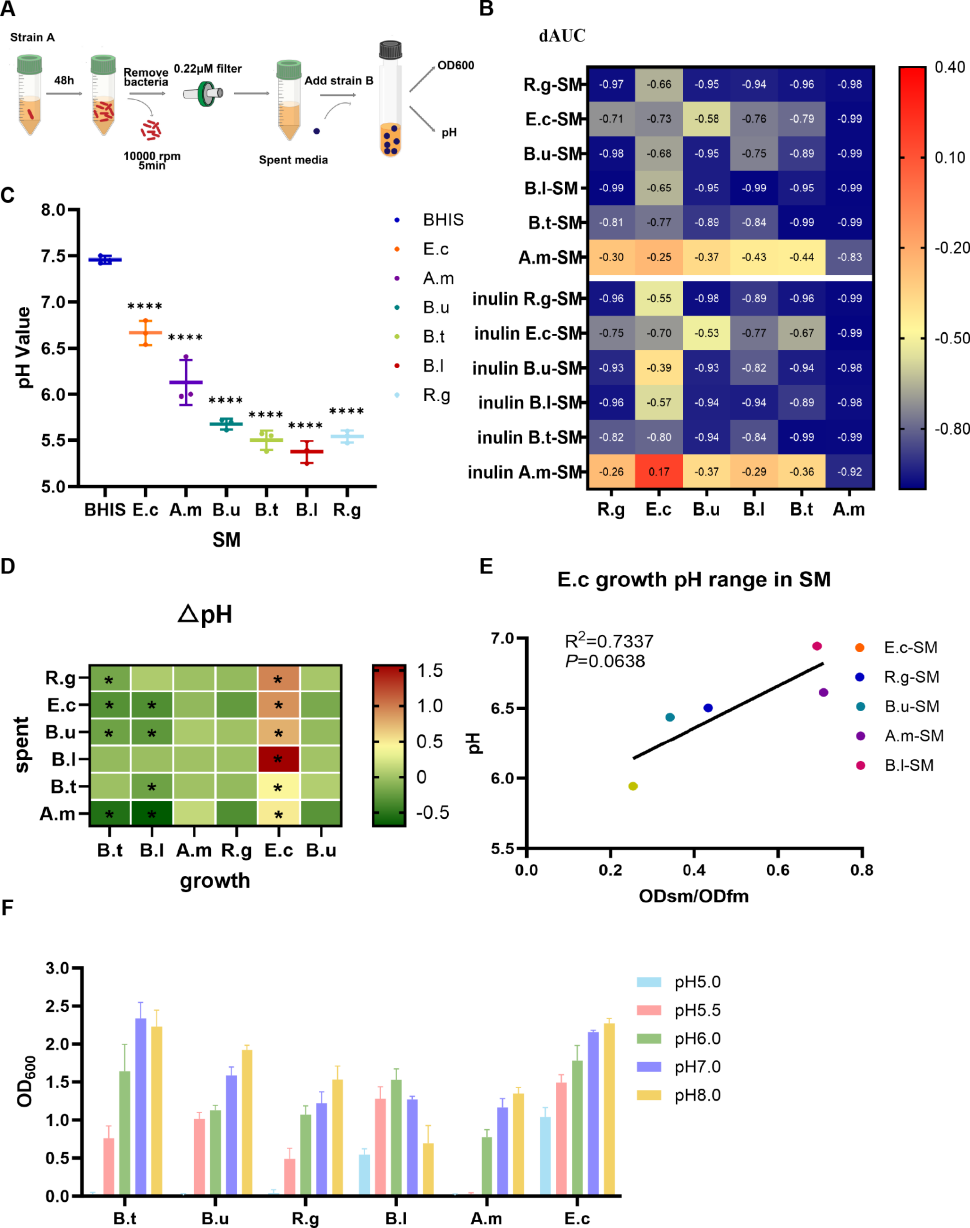
Bacterial interactions based on spent culture media experiments. (**A**) Flowchart depicting spent medium (SM) preparation in BHIS. (**B**) Monoculture growth in SM was analyzed by calculating the inhibition factor dAUC. dAUC was calculated from the mean AUC of three independent experiments relative to the mean AUC in fresh medium. (**C**) The mean pH of all SM after growth of the individual strains in BHIS medium. The respective SM was determined from three independent experiments (*n* = 3). Statistical analysis was performed using one-way ANOVA with Dunnett’s post-hoc test against the BHIS control. ****P < 0.0001. (**D**) The pH changes before and after culturing bacteria in SM of each strain. *P < 0.05. Each value is from at least three independent biological replicates. (**E**) The correlation between the *E. coli* dSM pH and OD600 ratio of *E. coli* grown in spent medium of each bacterium and fresh medium. (**F**) Bacterial growth in BHIS medium with different pH values. Data are presented as mean ± SD from three independent experiments.

A spent medium (SM) experiment was performed to explore the bacterial interaction dynamics under nutrient-rich conditions (Fig. 4A). A normalized inhibition factor (dAUC) was used to determine bacterial interaction patterns (22). As shown in Fig. 4B, when cultured in self-conditioned SM, all strains exhibited reduced growth (dAUC <−0.5), indicating that the pre-cultured medium no longer supported optimal growth, likely due to nutrient depletion. All strains showed strong inhibitory effects on the growth of the other bacteria, except *A. muciniphila* SM, which stimulated the growth of all five other bacteria (Fig. 4B). Overall, the inhibitory effects of all SM on *E. coli* were relatively weak. Upon supplementation of the BHIS medium with the prebiotic inulin (0.5%), inhibition indices between bacteria showed a slight trend toward less negative values, suggesting a modest attenuation of inhibitory effects, although the overall interaction patterns remained similar to those observed in BHIS alone (Fig. 4B). Notably, a weak trend toward mutualism (+/+) emerged between *E. coli* and *B. uniformis*. These findings suggest that while bacteria generally exhibit negative interactions under nutrient-rich conditions, the addition of prebiotics may mitigate this competitive pressure.

Microbial interactions are often determined by how bacterial metabolism alters the environment and how bacteria respond to these changes. A key environmental parameter is pH. We measured the pH values of SM and dSM (double-spent medium). Growth of *B. thetaiotaomicron*, *B. uniformis*, *B. longum*, and *R. gnavus* significantly acidified the environment, with *B. longum* and *B. thetaiotaomicron* exhibiting the most pronounced effects (Fig. 4C). Although pH in *E. coli* and *A. muciniphila* cultures was significantly lower than the initial pH, it remained slightly higher than that of the other strains (Fig. 4C). Cultivation of bacteria in SM of other bacteria revealed that *E. coli* could grow in SM of all other strains and significantly alkalized their pH (Fig. 4D). In contrast, *B. thetaiotaomicron* and *B. longum* substantially acidified the SM of most other strains (Fig. 4D). Correlation analysis revealed a strong but statistically non-significant relationship between pH and the OD ratio (OD_spent media_/OD_fresh media_) for *E. coli* (*R* = 0.7337, *P* = 0.0638), but not for *B. longum* and *B. thetaiotaomicron* (Fig. 4E; Fig. S4A and B). Bacterial growth assays across a pH gradient revealed that *E. coli* is tolerant to both acidic and alkaline pH conditions, whereas *B. longum* exhibits a strong tolerance to acidic environments (Fig. 4F). These results suggest that *E. coli* may enhance its own growth by modifying environmental pH, and that the pronounced inhibitory impact of *B. longum* on other bacteria in co-culture systems may be attributed to its strong acidification of the environment.

### Community structure of the synthetic microbiome in different nutrient conditions

**Fig 5:**
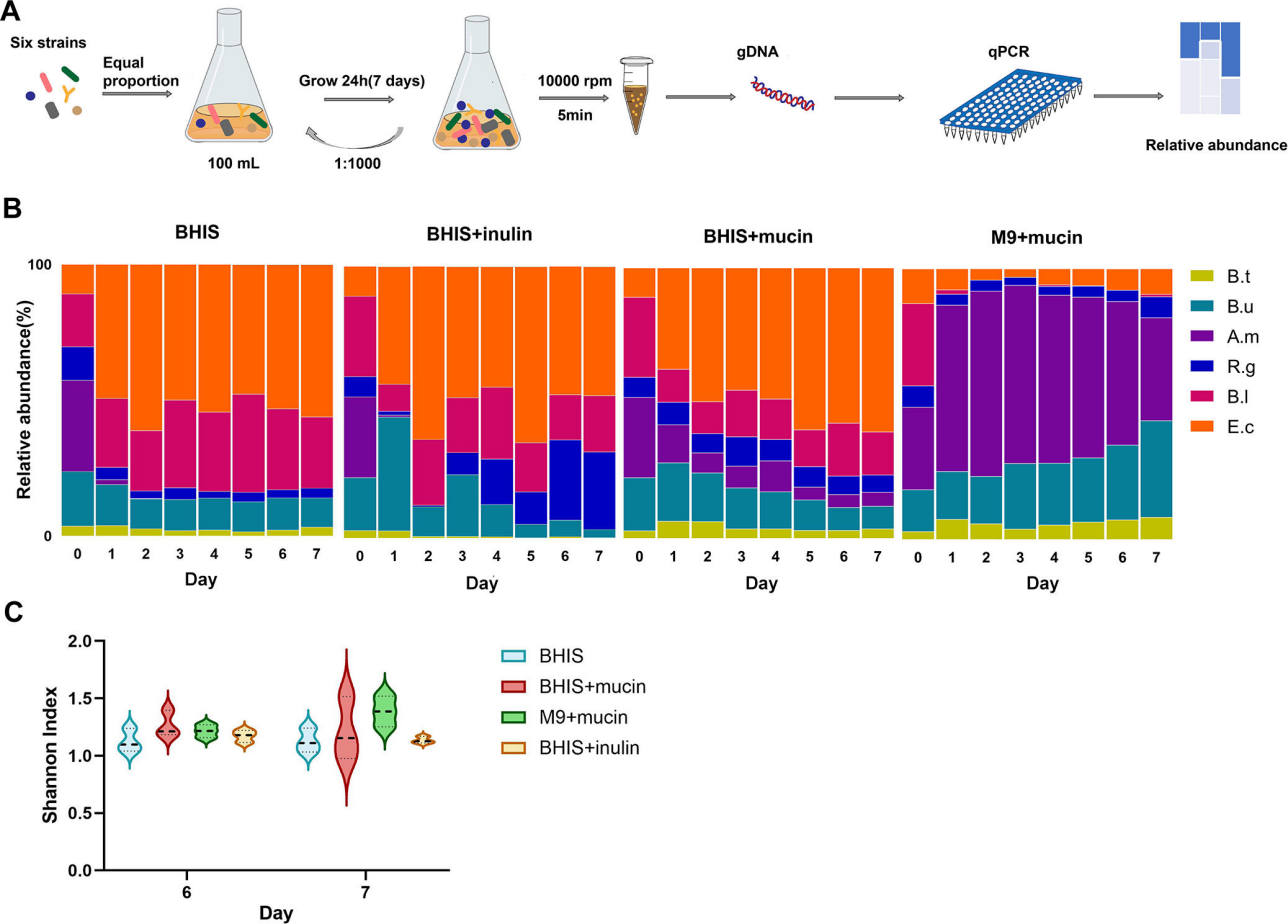
Influence of different nutritional conditions on the structure of the synthetic community. (**A**) Flowchart depicting *in vitro* bacterial abundance dynamics in community culture. (**B**) Bacterial abundance in the community was determined by qPCR after 7 days of serial passaging from three independent experiments in BHIS, BHIS supplemented with 0.5% inulin, BHIS supplemented with 0.5% mucin, and DMM supplemented with 0.5% mucin. (**C**) Shannon index of microbial community at different nutrient conditions at days 6 and 7 (*n* = 3). Shannon index was calculated with the following formula: *H* = −∑[(pi) × log(pi)]. *H* is the Shannon index and pi indicates the proportion of individuals of the *i*-th species in the whole community. One-way ANOVA showed no significant differences among groups at either time point (*P* > 0.05).

To investigate bacterial interaction patterns within a community context, we assembled a simplified synthetic community by mixing the bacterial strains. This community was cultured under various conditions, with daily 1:100 serial passages into fresh medium for 7 days (Fig. 5A). Quantitative PCR (qPCR) was used to monitor dynamic changes in the relative abundance of each bacterium. In BHIS medium, *E. coli*, a fast-growing strain, rapidly dominated the community, while *A. muciniphila* abundance declined from day 3 onwards (<0.05%) (Fig. 5B). The remaining strains maintained stable relative abundances throughout the 7-day period (Fig. 5B). Inulin supplementation in BHIS medium significantly inhibited *B. uniformis*, *B. thetaiotaomicron*, and *A. muciniphila*, while promoting *R. gnavus* (Fig. 5B). Although inulin is considered a prebiotic that can enhance microbial diversity, it did not substantially alter the overall community diversity in this context. The number of strains with >0.5% relative abundance was reduced to 4 by day 5 (Fig. 5B). Mucin supplementation in BHIS medium decreased *B. uniformis*, *B. thetaiotaomicron*, and *A. muciniphila*, but their abundances stabilized from day 5 onward, and all six strains maintained relative abundances above 3% (Fig. 5B). In DMM with mucin as the sole carbon source, the relative abundance of all community members remained above 0.5% throughout the experiment (Fig. 5B). Shannon diversity indices calculated at days 6 and 7 revealed that mucin supplementation promoted the maintenance of all community members in the BHIS, whereas the effect of inulin was less pronounced, although differences were not statistically significant (Fig. 5C). To evaluate the effect of mucin-degrading strains in DMM with mucin, we performed drop-out experiments. Removing *B. uniformis*, *B. thetaiotaomicron*, or *A. muciniphila* individually did not affect the overall community structure, suggesting potential functional redundancy or overlapping roles among these three strains in maintaining the structural integrity of the synthetic community (Fig. S5).

### Effect of mucin on the gut microbiome *in vivo*

**Fig 6:**
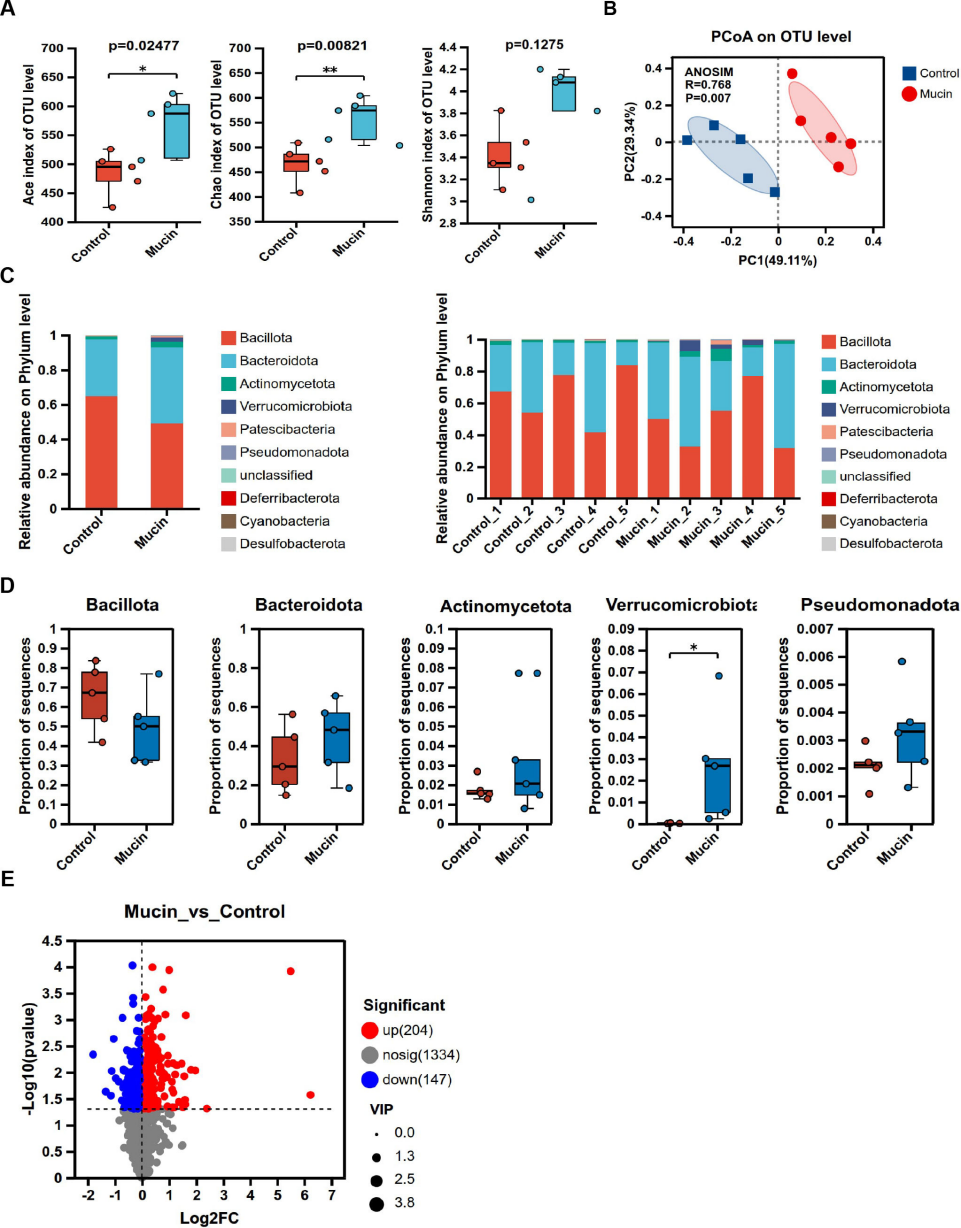
Dietary mucin alters gut microbiota structure and metabolic function. (**A**) Alpha diversity metrics of gut microbiota in mucin-treated vs control mice, assessed by ACE, Chao1, and Shannon indices (*P < 0.05 and **P < 0.01, Wilcoxon rank-sum test). (**B**) PCoA of gut microbiota at OTU level (ANOSIM, *R* = 0.7680, *P* = 0.007). (**C**) Relative abundance of gut microbiota at the phylum level in the two groups of mice. The left panel shows the comparison of mean relative abundance between the two groups. The *x*-axis represents the groups, and the *y*-axis represents the relative abundance (%). Data are presented as mean values. The right panel shows the relative abundance distribution for individual mice. (**D**) Abundance of five main phyla in gut microbiome of two groups (*P < 0.05, Wilcoxon rank-sum test). (**E**) The volcano plot highlights significantly differential metabolites between the two groups. The *x*-axis represents the log_2_ fold change, while the *y*-axis represents the −log_10_ adjusted *P* value.

To evaluate the *in vivo* effects of mucin on the gut microbiota, five age-matched female C57B/L6 mice were given 1% mucin in the drinking water for 1 month, while five control mice received normal drinking water. 16S rRNA sequencing revealed that mucin supplementation significantly increased alpha diversity indices in the stool compared to the controls (Fig. 6A). Principal coordinate analysis (PCoA) showed significant separation between the two groups, indicating that mucin substantially impacted gut microbiota composition (Fig. 6B). At the phylum level, mucin significantly increased the abundance of Verrucomicrobiota, Bacteroidota, and Actinomycetota, while decreasing Bacillota (Fig. 6C and D). The abundance of Pseudomonadota remained largely unchanged (Fig. 6D). Consistent with these phylum-level shifts, genus-level analysis revealed that mucin supplementation notably increased the relative abundance of *Akkermansia* (Verrucomicrobiota), *Bacteroides*, and Prevotellaceae (Bacteroidota), and *Bifidobacterium* (Actinomycetota), while decreasing multiple genera within Bacillota, including *Lactobacillus*, Lachnospiraceae NK4A136 group, and *Clostridium sensu stricto 1* (Fig. S6). Gut microbiota co-occurrence network analysis suggested that mucin administration was associated with an increase in network nodes and edges, which may reflect a higher frequency of potential bacterial co-occurrence patterns (Fig. S7). The proportion of positively correlated edges was slightly increased, while negatively correlated edges decreased, which is consistent with the possibility of enhanced cooperation and reduced competition under the tested conditions (Fig. S7). Untargeted LC-MS metabolomics analysis identified 349 significantly dysregulated metabolites between the two groups. Classification by biological roles showed that phospholipids represented the most abundant category, followed by steroids and vitamins/cofactors, whereas nucleotides and oligosaccharides were only minimally represented (Fig. 6E; Fig. S8). KEGG pathway analysis revealed enrichment in lipid metabolism, biosynthesis of secondary metabolites, amino acid metabolism, and microbial cofactor metabolism (Fig. S8). Correlation analysis between the relative abundances of five bacterial genera and the top 50 differential metabolites revealed highly similar metabolic profiles for *Akkermansia* and *Bacteroides*, as well as for *Ruminococcus* (Fig. S10). In contrast, no significant correlation was observed between the metabolites and those of *Escherichia* or *Bifidobacterium*.

## DISCUSSION

The human colon serves as a major habitat for bacteria, with the mucosal layer being the primary site of colonization (8). Beyond dietary nutrients, host-derived mucin represents a crucial nutrient source for the gut microbiota (23). Given the vast diversity and complexity of the gut microbiota and its nutritional environment, we employed an *in vitro* approach in this study to study the interaction between bacteria in mucus. We constructed a simplified synthetic community composed of human gut isolates and performed monoculture, pairwise co-culture, and community culture with 16S rRNA gene sequencing and metabolomics analyses to investigate the interaction patterns of mucosal-associated bacteria. Our findings reveal that these bacteria exhibit increased negative interactions under nutrient-rich conditions and a greater propensity for positive interactions under nutrient-poor conditions. The presence of mucin was associated with fewer negative interactions and a higher proportion of positive interactions under both low- and high-nutrient conditions. Furthermore, our *in vivo* data suggest that mucin supplementation is consistent with the promotion of positive interactions among gut microbes and increased community diversity.

It is well-established that diet is a crucial determinant of gut microbiota composition, with host dietary intake significantly altering bacterial interaction patterns (24, 25). The nutrient availability within the intestinal environment directly influences interspecies bacterial interactions and community structure (26). In our study, a DMM supplemented with mucin was employed to simulate a nutrient-depleted intestinal environment where mucin served as the sole carbon source. Under these conditions, we observed a preponderance of positive interactions, which are consistent with cross-feeding by mucin-degrading bacteria that provide metabolites for other species. Specifically, *A. muciniphila* is known to cleave terminal sugars such as sialic acid and fucose, but does not efficiently consume these released monosaccharides, suggesting that these sugars may become available for other community members. This is consistent with the possibility that *A. muciniphila* promotes the growth of other species (27, 28). In addition to these liberated monosaccharides, other metabolic intermediates generated during mucin fermentation, such as acetate, propionate, succinate, and 1,2-propanediol could also be involved in the observed positive interactions (27, 29, 30). For example, succinate, another common intermediate generated by *Bacteroides* species during glycan fermentation, serves as a precursor for propionate production via the succinate pathway (29). Additionally, fucose liberated from mucin glycans can be metabolized via the 1,2-propanediol pathway, yielding propionate and 1,2-propanediol as intermediates that support specific cross-feeding partnerships (30). Thus, the positive interactions observed in our mucin-containing communities are likely mediated by a combination of these metabolic intermediates, ultimately contributing to the maintenance of a stable community structure under mucin-rich, nutrient-limited conditions. Conversely, BHIS medium, representing a nutrient-rich environment, exhibited a significant increase in negative interactions, which finally led to a reduction in species richness (26). Furthermore, in co-culture and *in vitro* community experiments, we found that supplementing BHIS with either inulin or mucin promoted microbial richness. However, inulin exhibited a weaker stabilizing effect on community structure compared to mucin, which more effectively maintained both stability and diversity. Therefore, *in vitro* mucin supplementation may represent a promising strategy for preserving community stability and enhancing microbial richness.

pH is a crucial environmental factor influencing microbial physiology and community dynamics (31). Certain bacteria, such as *Bifidobacteria*, can acidify the surroundings through the production of lactic acid and acetate, thereby altering the environment and impacting the growth and survival of coexisting species (32). These pH alterations can also shift interaction patterns within the bacterial niche (31, 33). Our SM pH measurements revealed that the *B. longum* SM exhibited the lowest pH across all tested bacteria. Furthermore, cultivating *B. longum* in other bacterial SM demonstrated a significant acidification effect on the SM of four out of five bacterial species. Pairwise interaction assays indicated that *B. longum* significantly inhibited the growth of other bacteria across various nutrient conditions. Although no significant correlation was observed between dAUC and pH, the acidification of the environment by *B. longum* may contribute to its inhibitory effects on other species.

In animal experiments, we correlated mouse fecal metabolites with genus-level bacterial abundances. *Akkermansia* and *Bacteroides*, two known mucin-degrading genera, were associated with a largely overlapping set of mucin-induced metabolites. This functional redundancy in mucin degradation is consistent with our *in vitro* findings that removing any single mucin-degrading member did not significantly alter the overall community structure (Fig. S4). Although *R. gnavus* has been reported to degrade mucin (34), our experiments did not confirm this capability using one strain. However, metabolite correlation analysis demonstrated a close resemblance between *Ruminococcus* and the other three mucin-degrading genera, albeit not identical, suggesting its association with mucin degradation pathways.

It is important to acknowledge a key limitation of our experimental model. This study utilized commercially available porcine gastric mucin, which is primarily composed of MUC5AC and MUC6—the major mucins secreted by the gastric epithelium. In contrast, the human colonic mucus layer is predominantly formed by MUC2, a structurally and functionally distinct mucin. Beyond differences in the protein backbone, gastric and colonic mucins exhibit substantial variations in glycosylation patterns, including the density and branching of O-glycans, as well as the degree and linkage of terminal modifications such as sialylation and fucosylation (35). These structural differences may influence microbial adhesion, enzymatic degradation, and the repertoire of glycans available for cross-feeding. Furthermore, commercially prepared mucin preparations are known to contain contaminating low-molecular-weight nutrients, which may independently influence bacterial phenotypes. Nevertheless, porcine gastric mucin remains a convenient and widely used model substrate. Future studies using colonic mucin or more physiologically relevant models would help validate the interaction patterns observed here.

Our study presents a comprehensive investigation of mucosal-associated bacterial strain-strain interaction in varying nutritional conditions. This work provides a foundation for understanding *in vivo* mucus dynamics and developing targeted microbiome interventions to improve human health.

## MATERIALS AND METHODS

### Identification of core gut bacterial species

To identify core health-associated gut bacterial species, we retrieved taxonomic abundance data from the GMrepo v2 database (https://gmrepo.humangut.info) (17), which aggregates publicly available human gut metagenomic data sets. From this database, we initially selected all species with a relative abundance >0.5% in healthy individuals (data points shown in Fig. 1A). From this initial set, we further filtered for species with a prevalence >25% across the healthy population, yielding 56 core species (Table S1). The six bacterial strains used in this study were selected from these 56 core species, representing the five dominant phyla of the human gut microbiota. All selected strains have been reported to possess mucin-degrading potential or to serve as dominant colonizers of the human colonic mucus layer. Specifically, two Bacteroides species (*B. thetaiotaomicron* and *B. uniformis*) were included to capture potential functional redundancy or divergence within this dominant mucin-degrading genus.

### Bacterial strain isolation and growth condition

Stool sample was cultured on selective culture medium including *Bacteroides* bile esculin (BBE) plate (*B. uniformis*), lactose broth agar plate (*E. coli*), *Bifidobacterium* agar (*B. longum*), and defined minimal medium with mucin plates (*A. muciniphila*) for the first step screening. Colony PCR with species-specific primers was performed for further screening. Final validation was conducted by 16S rRNA sequencing and whole genome next-generation sequencing. Sequences of all primers used in this study are listed in Table S6. The bacterial strains used in this study, including their sources and GenBank accession numbers, are listed in Table S2.

All strains were grown in brain heart infusion supplemented with 5 mg/L hemin and 2.5 µg/L vitamin K1 (BHIS) or DMM medium (Table S3) with or without 1.5% agar. Depending on the experimental condition, mucin, glucose, and inulin were added at a final concentration of 0.5%. Antibiotics concentrations used for selection are as follows: gentamicin 100 mg/L, erythromycin 10 mg/L, and polymyxin B 2 mg/L. Cultures were incubated at 37°C without shaking under strictly anaerobic conditions (gas atmosphere 7% H_2_, 10% CO_2_, and 83% N_2_).

### Bacterial growth measurements

For most conditions, the initial inoculum for all experiments was prepared by resuspending an overnight culture in PBS and adjusting the OD_600_ to 1.0. The inoculum was then transferred into fresh medium to achieve a starting OD_600_ of 0.01, except for experiments performed in DMM, where the starting OD_600_ was adjusted to 0.1. The growth of bacterial co-cultures and monocultures in media supplemented with mucin (Type III, Sigma-Aldrich, USA; cat. no. M1778, lot no. SLCN1002) was evaluated using serial dilution for CFU counting alongside their distinct antibiotic susceptibility profiles or colony morphologies.

### Determination of bacterial co-culture outcomes

The co-culture outcomes, classified as positive (+), neutral (0), or negative (−), were determined by calculating the CFU ratio for each strain in co-culture relative to monoculture. Therefore, the CFU in all pairwise co-cultures was divided by the CFU in monoculture at 48 h time point $\left(r=\frac{{CFU}_{\text{monoculture}}(48h)}{{CFU}_{\text{coculture}}(48h)}\right)$. The mean CFU ratio from all individual experiments (*n* = 3 per strain combination) was calculated, and *P* values were determined using a Student’s *t*-test.

### Phylogenetic tree construction

The phylogenetic tree based on the comparison of whole genomes of the six strains was constructed using CVTree v3 with default parameters. The resulting tree file was then uploaded to MEGA X for visualization.

### Analysis of bacterial genomic potential for mucin degradation

Bacterial glycosyl hydrolase (GH) families involved in mucin degradation were examined as previously described (20). In brief, GH genes were retrieved from the Carbohydrate-Active enZYmes (CAZy) database (https://www.cazy.org), and bacterial GH gene copy numbers were subsequently determined from annotated genomes. KEGG functional annotation for each bacterial species was performed using eggNOG-Mapper based on their respective genomes. Subsequently, KEGG pathway information specifically pertaining to the five monosaccharide components of mucin O-glycan—L-fucose (Fuc), D-galactose (Gal), N-acetyl-D-galactosamine (GalNAc), N-acetyl-D-glucosamine (GlcNAc), and N-acetylneuraminic acid (Neu5Ac)—was extracted to facilitate functional prediction (36).

### Generation of spent culture media

Bacterial subcultures were cultivated for 24 h in 10 mL of BHIS broth, either with or without inulin supplementation, under anaerobic conditions at 37°C without shaking. Subsequently, culture supernatants (SM) were harvested from the resulting subcultures by centrifugation at 10000 × *g* for 5 min, followed by pH assessment and sterilization using a 0.22-µm filter. Under anaerobic conditions, the bacterial monoculture was inoculated into the SM with the initial inoculum size as described previously. The OD_600_ and pH were measured at 48 h time point.

### Quantitative PCR of bacterial strains

The relative abundance of all species in the synthetic community under various conditions was determined by qPCR. Total DNA from the microbial community was extracted using the GeneJET Genomic DNA Purification Kit (Thermo Scientific). The DNA concentration was measured using a Nucleic Acid and Protein Analyzer (Nano-200). Five nanograms of gDNA was used as a template for qPCR. Standard curves of each bacterial strain were generated at six standard concentrations, ranging from 0.1 pg to 10 ng/µL (Fig. S9). qPCR measurements were performed in triplicate in 10 μL reactions using the SYBR Green Realtime PCR Master Mix. The thermal cycling conditions were as follows: 95°C for 1 min, 40 cycles at 95°C for 15 s, 60°C for 15 s, and 72°C for 45 s.

### *In vitro* community experiments

OD_600_ of monoculture inocula of each strain was adjusted to 1.0 and mixed in approximately equal proportions and inoculated into 100 mL of fresh medium at a ratio of 1:100. The community inoculum was incubated at 37°C without shaking under anaerobic conditions with daily 1:100 serial passages into fresh media for 7 days. Samples were taken every 24 h for qPCR analysis. The number of bacteria in the community was calculated per ng of DNA according to the formula: number of copies = (amount of amplicon [ng] × 6.0221 × 10^23^ molecules/mol)/(average genome size × 660 g/mol) × 1 × 10^9^ ng/g.

### Animal experiments

Ten age- and body weight-matched (6–8 weeks old) female C57BL/6 mice (Charles River Laboratories Inc., Beijing, China) were divided into two experimental groups. All mice were bred and maintained under specific pathogen-free conditions, with free access to autoclaved water and sterile food. The treatment group received 1% mucin supplementation in their drinking water for 1 month, while the control group received normal drinking water. After 1 month, mouse stool samples were collected for 16S rRNA sequencing and gut metabolites profiling using untargeted liquid chromatography-mass spectrometry (LC-MS).

### Untargeted LC-MS/MS metabolomics

Fecal samples (50 mg) were homogenized with 400 μL of extraction solution (methanol:water = 4:1, vol:vol) containing 0.02 mg/mL internal standard (L-2-chlorophenylalanine) using a frozen tissue grinder (Wonbio-96c; −10°C, 50 Hz) for 6 min. After ultrasonic extraction (30 min, 5°C, 40 kHz) and incubation at −20°C for 30 min, the mixture was centrifuged (13,000 × *g*, 15 min, 4°C). The supernatant was collected for LC-MS/MS analysis.

Metabolomic profiling was performed on a Thermo Scientific UHPLC-Q Exactive HF-X system (Thermo Fisher Scientific, USA) equipped with an ACQUITY UPLC HSS T3 column (100 mm × 2.1 mm, 1.8 μm; Waters, USA). Chromatographic separation was performed using solvent A (0.1% formic acid in water/acetonitrile, 95:5, vol/vol) and solvent B (0.1% formic acid in acetonitrile/isopropanol/water, 47.5:47.5:5, vol/vol/vol) under the following gradient conditions: 0–3 min, 0–20% B; 3–4.5 min, 20–35% B; 4.5–6.3 min, 35–100% B; 6.3–8 min, 0% B, at a flow rate of 0.40 mL/min and a column temperature of 40°C. The mass spectrometer was operated in both positive and negative ESI modes using data-dependent acquisition with a full MS resolution of 60,000, MS/MS resolution of 7,500, normalized collision energies of 20/40/60 eV, and an *m/z* scan range of 70–1,050. Raw data were processed using Progenesis QI (Waters Corporation, Milford, USA). Following removal of internal standards and artifacts (noise, column bleed, and derivatized reagent), metabolites were annotated against HMDB (http://www.hmdb.ca/), METLIN (https://metlin.scripps.edu/), and Majorbio Database. Sum normalization was applied to correct for technical variability, and metabolic features with >30% relative standard deviation (RSD) in quality control samples were excluded prior to log_10_ transformation, generating the final data matrix for downstream statistical analysis. Metabolites with variable importance in the projection (VIP) >1.0 and false discovery rate (FDR)-adjusted *P* value < 0.05 (based on Student’s *t*-test) were considered significantly different metabolites. The data were analyzed through Majorbio Cloud platform (cloud.majorbio.com).

### Bacterial whole-genome sequencing and assembly

Chromosomal DNA was extracted using the FastPure Stool DNA Isolation Kit (MJYH, Shanghai, China) according to the manufacturer’s protocol. DNA purity and concentration were assessed using a NanoDrop 2000 and a Quantus Fluorometer (Picogreen). Genomic DNA was then sheared to ~400 bp fragments using a Covaris M220 Focused Acoustic Shearer. Sequencing libraries were prepared with the NEXTFLEX Rapid DNA-Seq Kit (Bioo Scientific, USA) following the manufacturer’s protocol. Paired-end sequencing (2 × 150 bp) was performed on an Illumina NovaSeq XPlus (Illumina Inc., San Diego, CA, USA) after PCR amplification of adapter-ligated fragments. Raw reads were quality-filtered using fastp (v0.19.6) with the following parameters: trimming low-quality bases (*Q* < 20), and discarding reads <30 bp. *De novo* genome assembly was performed with SOAPdenovo2 (v2.04) using optimized k-mer values (21, 33, 55, 77).

### 16S rRNA gene amplicon sequencing

Bacterial DNA was extracted from mouse fecal samples using the QIAamp Fast DNA Stool Kit (Qiagen). The hypervariable V3–V4 region of the bacterial 16S rRNA gene was amplified using primers 338F (5′-ACTCCTACGGGAGGCAGCAG-3′) and 806R (5′-GGACTACHVGGGTWTCTAAT-3′) on an ABI GeneAmp 9700 PCR Thermocycler (Applied Biosystems, Foster City, USA). Purified amplicons were pooled in equimolar amounts and paired-end sequenced on an Illumina MiSeq PE300 platform (Illumina, San Diego, USA) according to the standard protocols by Majorbio Bio-Pharm Technology Co. Ltd. (Shanghai, China). All of the above analyses were performed using the I-Sanger Cloud Platform (https://www.i-sanger.com/) from Shanghai Majorbio.

### Microbiota composition analysis

Gut microbiota bioinformatic analysis was performed using the Majorbio Cloud platform (https://cloud.majorbio.com). Alpha diversity indices (Ace, Shannon, and Chao indexes) were calculated at the operational taxonomic unit (OTU) level using Mothur (v1.30.2). Differences between groups were assessed using the Wilcoxon rank-sum test. Microbial community structure was visualized using principal coordinates analysis (PCoA) based on the Weighted UniFrac distance metric using R-3.3.1 Vegan package, and analysis of similarities (ANOSIM) was used to identify significant dissimilarities. Bacterial taxonomic profiling at the phylum level was compared among groups using the Wilcoxon rank-sum test.

### Network analysis

To investigate the differences in microbial community interactions between the control group and the mucin group, we established co-occurrence networks using Cytoscape v3.7.1 (37) and the CoNet v1.1.1 plugin (38). The rarefied OTU data were used as an input matrix. Within the CoNet plugin, we employed Pearson and Spearman correlation coefficients, Kullback-Leibler dissimilarity matrices, and mutual information to infer microbial interactions. The software calculates *P* values based on both permutation and bootstrap distributions.

### Mucin glycan purification

Porcine gastric mucin (10% wt/vol) was reduced with 150 mM NaOH and 750 mM NaBH_4_. Porcine gastric mucin (Sigma Type III, 10% wt/vol) was reduced for 20 h with 150 mM NaOH and 750 mM NaBH4 (48°C, 20 h), neutralized with 10 M HCl, and centrifuged (14,000 × *g*, 4 °C, 30 min) to remove insoluble material. The resulting supernatant was used for both glycan and protein backbone fragment preparations. For mucin glycan preparation, the resulting supernatant was dialyzed against dH_2_O using 1 kDa MWCO membranes and subsequently lyophilized. The lyophilized glycans were weighed and solubilized in dH_2_O to a final concentration of 0.4% (wt/vol) for use in the culture medium (39). To obtain deglycosylated mucin, the mucin solution (prepared at twice the standard experimental concentration, 1.0% wt/vol) was adjusted to a final concentration of 10% trichloroacetic acid (TCA) and incubated at 4°C for 1 h to precipitate the mucin. The precipitate was then collected by centrifugation at 14,000 × *g* for 30 min at 4°C, and the resulting pellet was washed with ice-cold acetone to remove residual TCA. The protein pellet was solubilized in dH_2_O. Deglycosylation efficiency was confirmed by a phenol–sulfuric acid assay, which detected no residual carbohydrate signal above background levels.

### Statistical analysis

GraphPad Prism (v9) was used for all statistics. Figures were partly generated using GraphPad Prism and Adobe Illustrator CC (Adobe Inc.). Data are presented as mean ± SD from at least three independent biological replicates. Normality was assessed using the Shapiro–Wilk test. If data were normally distributed, one-way analysis of variance (ANOVA) followed by Tukey’s multiple comparisons test was used for multi-group comparisons, and a two-tailed unpaired Student’s *t*-test was used for pairwise comparisons. Otherwise, nonparametric analyses were performed: the Mann–Whitney *U*-test for two groups, or the Kruskal–Wallis test followed by Dunn’s post hoc test for more than two groups. Differences were considered statistically significant at *P* < 0.05. Significance levels are indicated as *P < 0.05, **P < 0.01, and ***P < 0.001.

## Data Availability

The raw 16S rRNA sequencing data have been deposited in the NCBI Sequence Read Archive (SRA) under BioProject accession number PRJNA1136429. The raw whole-genome sequencing data of the four bacterial isolates are available under BioProject accession number PRJNA1306593. The assembled and annotated genome sequences for these isolates are available in the NCBI GenBank database under accession numbers JBGNFQ000000000.1, JBGNFR000000000.1, JBGNWW000000000.1, and JBGNWX000000000.1.

## SUPPLEMENTAL MATERIAL

Supplemental figures msphere.00289-26-s0001.docx Fig. S1 to S10.

Table S1 msphere.00289-26-s0002.xlsx Core species of human gut microbiome.

Table S2 msphere.00289-26-s0003.xlsx Information of synthetic community members.

Table S3 msphere.00289-26-s0004.xlsx Recipe of growth media used to grow members of synthetic microbiota.

Table S4 msphere.00289-26-s0005.xlsx Antibiotics used for bacterial selection in co-culture experiments.

Table S5 msphere.00289-26-s0006.xlsx MICs of different antibiotics.

Table S6 msphere.00289-26-s0007.xlsx Primers used for conventional PCR and qPCR in this study.

ASM does not own the copyrights to Supplemental Material that may be linked to, or accessed through, an article. The authors have granted ASM a non-exclusive, world-wide license to publish the Supplemental Material files. Please contact the corresponding author directly for reuse.
