# Supplementary material for: Deciphering the in vitro mucin-driven interaction dynamics of a synthetic gut bacterial community
Source: mSphere. 2026 Jul 10;11(7):e00289-26. doi: 10.1128/msphere.00289-26 (PMC13410983; doi:10.1128/msphere.00289-26)
Supplement: Supplemental figures — Fig. S1 to S10. [file msphere.00289-26-s0001.docx]

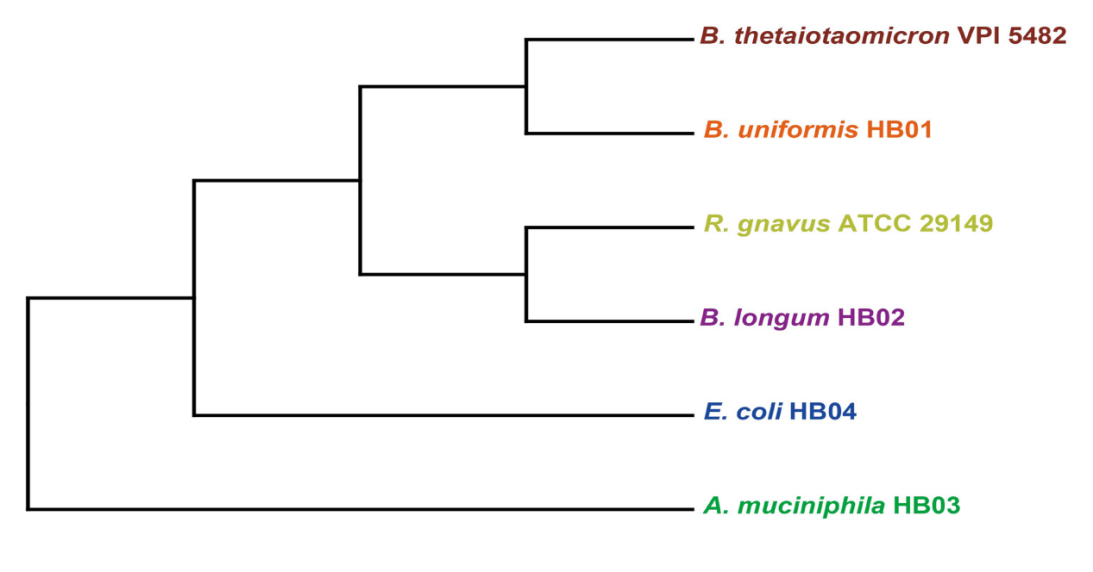


**Figure. S1 Phylogenetic tree of 6 synthetic community members based on the whole-genome sequences.** The phylogenetic tree shows the evolutionary relationships among the six bacterial strains used in the synthetic community, inferred from whole-genome sequences. The scale bar indicates genetic distance.


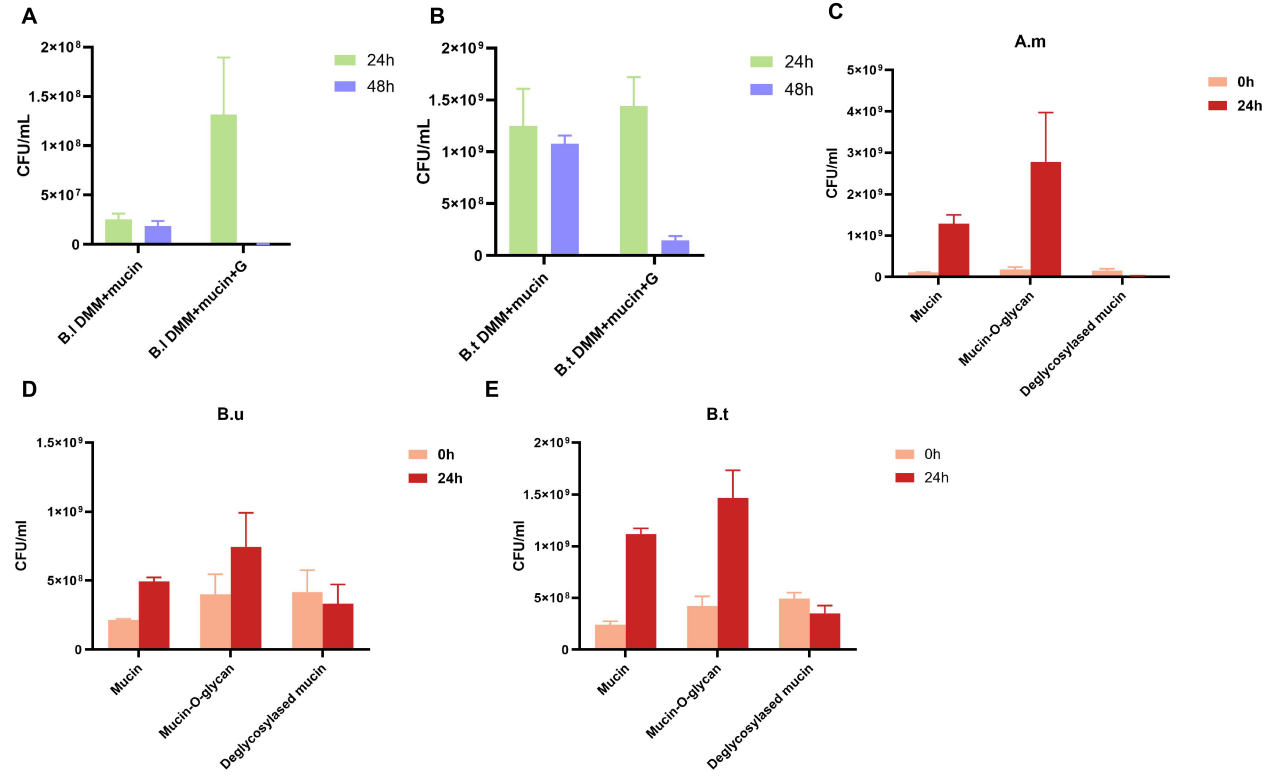


**Figure. S2 Bacterial growth in different nutrient condition.** Effect of glucose supplementation in DMM with mucin on growth of *B. longum* (A) and *B. thetaiotaomicron* (B) at 24h and 48h time points. Growth of *A. muciniphila* (C)*,* *B. uniformis* (D) and *B. thetaiotaomicron* (E) in DMM with purified mucin glycan or deglycosylated mucin as sole carbon source.


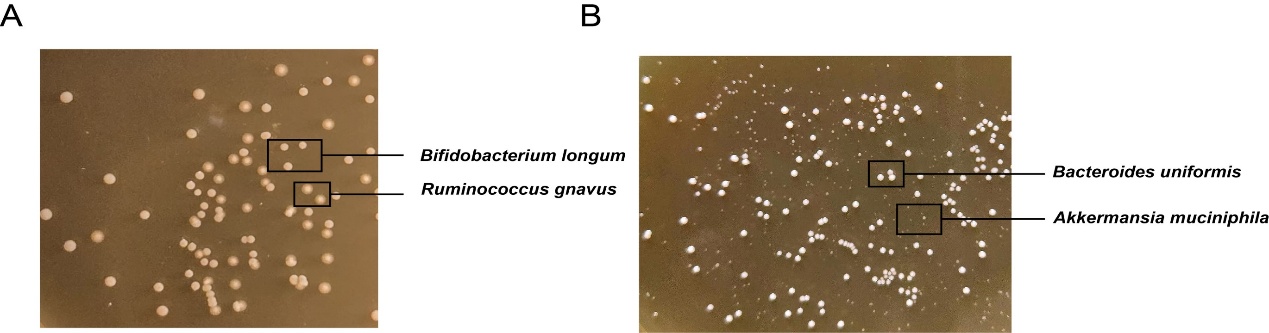


**Figure. S3** **Bacterial colony morphology on BHIS agar plate**. (A) Colony morphology of *B. longum* and *R. gnavus* coculture on same plate. (B) Colony morphology of *B. uniformis* and *A. muciniphila* coculture on same plate.


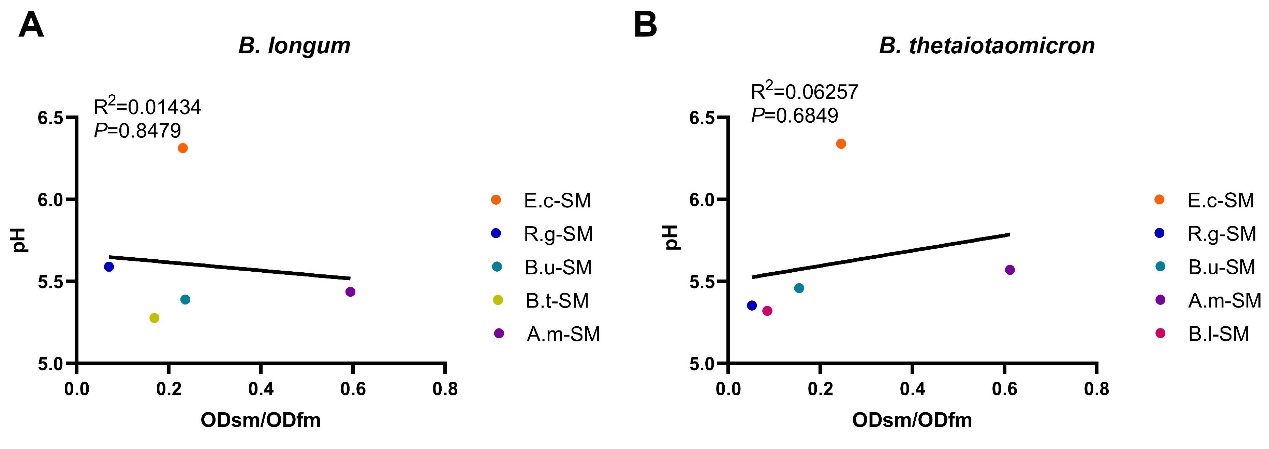


**Figure. S4 The correlation between the environmental PH and bacteria growth.** The correlation between the dSM PH and OD600 ratio (OD _spent media_/ OD_fresh media_) of *B. longum* (A) and *B. thetaiotaomicron* (B). The OD600 at 24h time point was monitored.


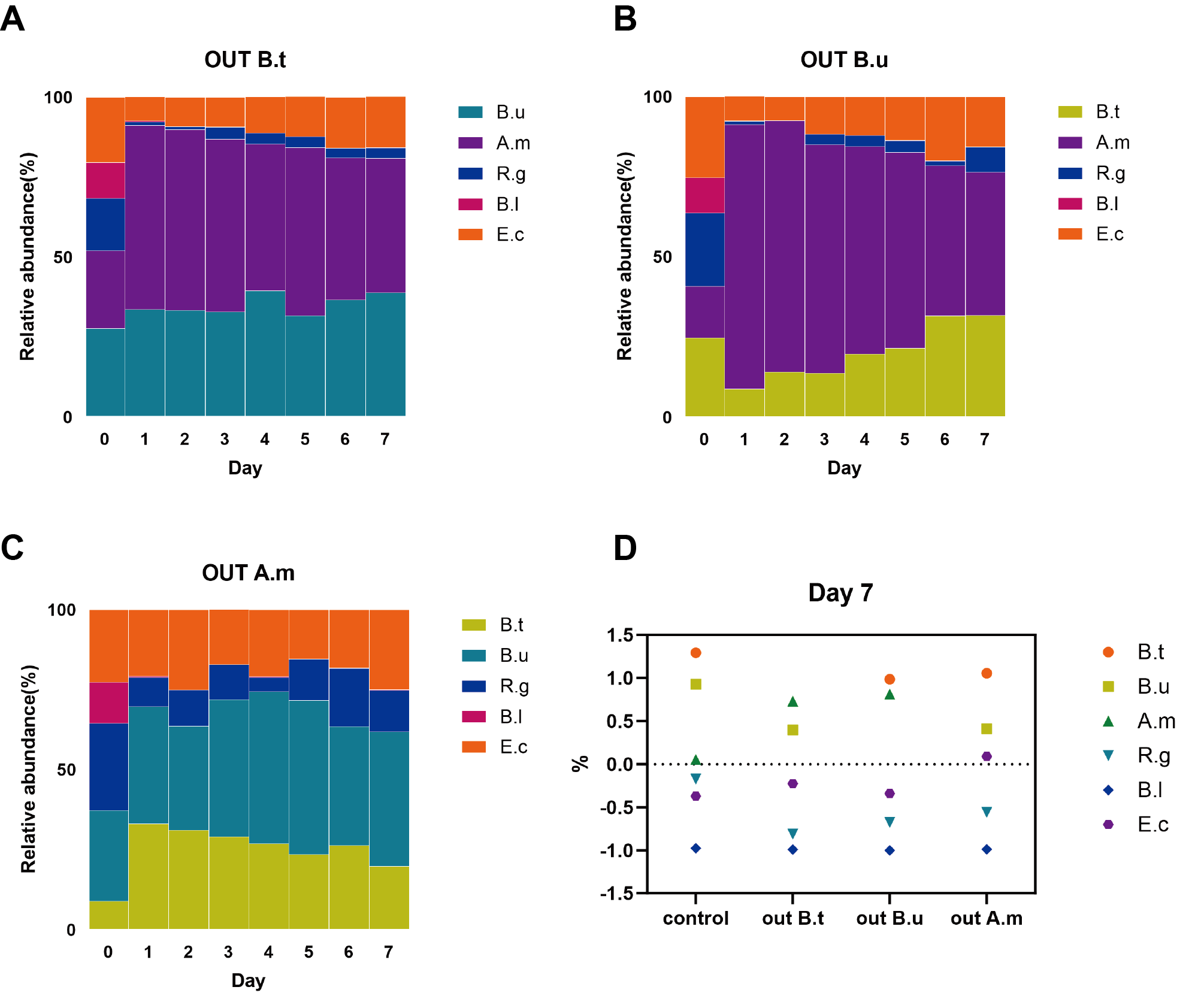


**Figure. S5 Effects of single strain drop-outs on the community structure.** Bacterial abundance in community was determined by qPCR after for seven days from three independent experiments in DMM with mucin after removing (A) *B. thetaiotaomicron*, (B) *B. uniformis* and (C) *A. muciniphila* respectively. (D) The effect of different bacterial dropout on the percentage change in community abundance between day 0 and day 7.





**Figure. S6 Relative abundance of gut microbiota at the genus level in the two groups of mice.** Comparison of relative abundance at the genus level between the Control group and the Mucin group. The top 15 genera in terms of abundance are displayed, with the remaining genera categorized as "others". Data are shown as mean values.


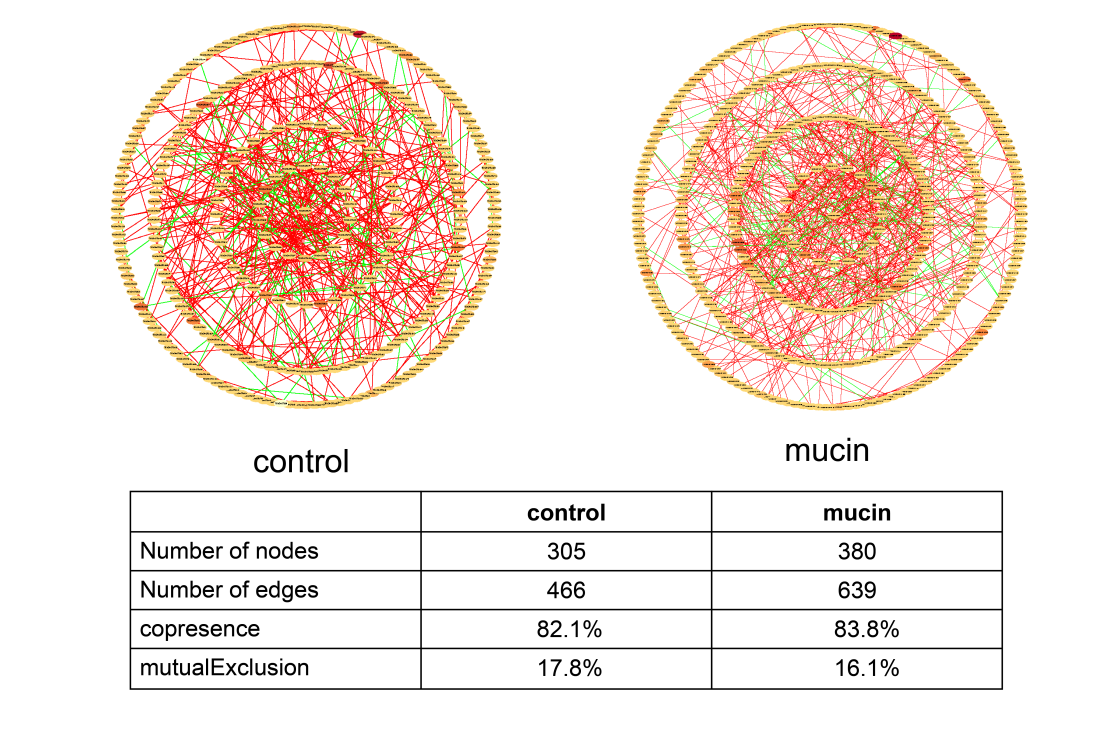


**Figure. S7** **Microbial interaction network in control and mucin groups.** Nodes represent OTUs and darker colors indicating higher abundance. The red edges indicating co-presence and green edges indicating mutual exclusion in the network. The higher the degree value, the closer the node is to the center of the circle.


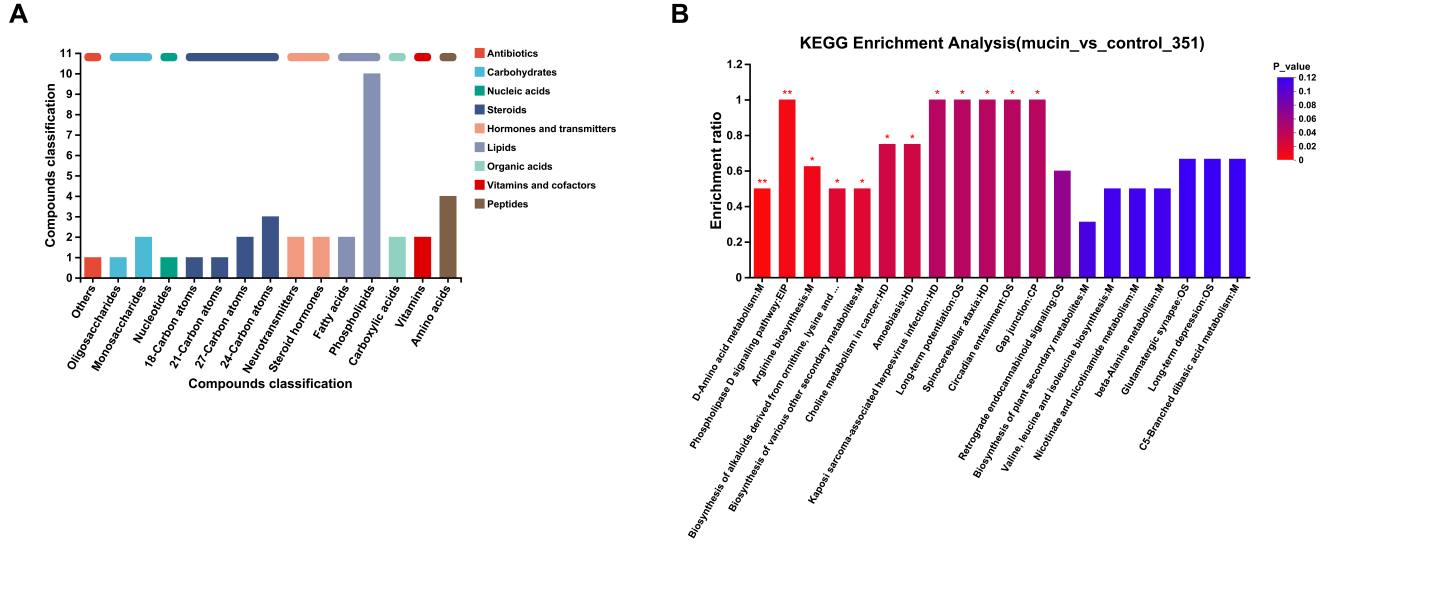


**Figure. S8 KEGG compound classification and pathway enrichment analysis of differential metabolites.** (A) Classification of metabolites based on annotations from the KEGG compound database. The bar chart displays the enrichment scores of differential metabolite subclasses across various compound categories; higher values indicate greater enrichment. (B) KEGG pathway enrichment analysis (top 20 pathways). Bar height represents the number of enriched metabolites, and color gradient indicates significance level (*p < 0.05; **p < 0.01).


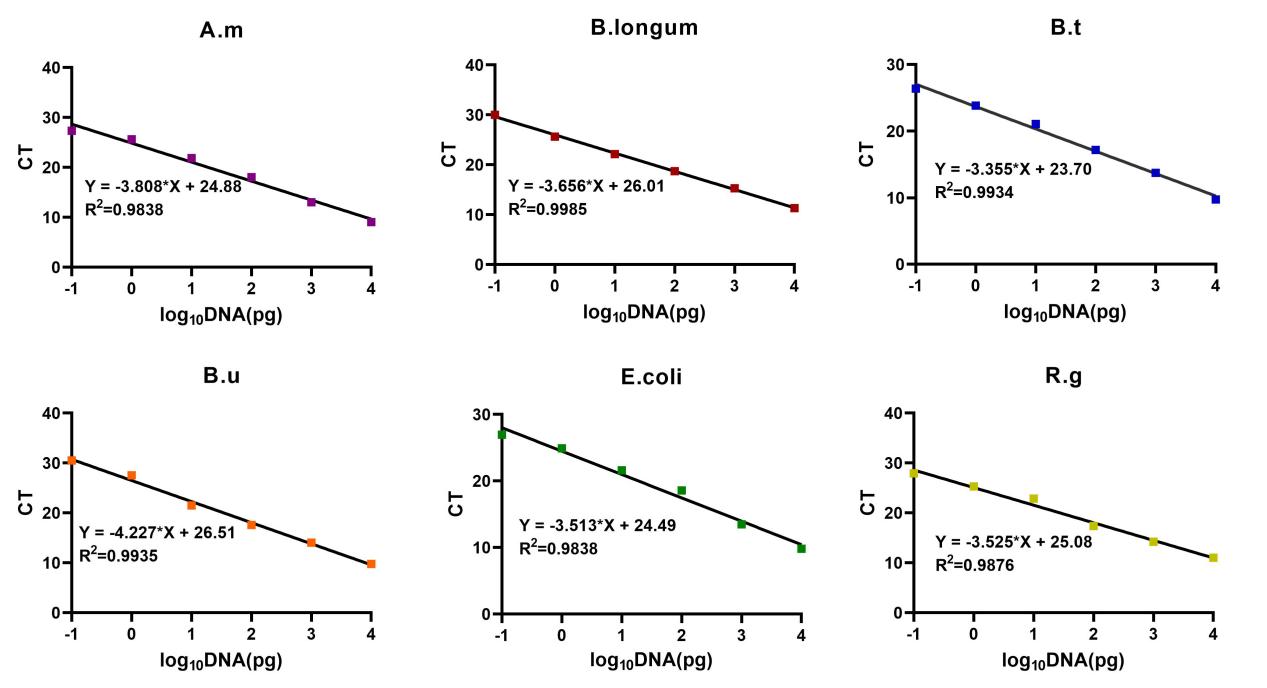


**Figure. S9 Standard Curve for qPCR Analysis.** The graph illustrates the correlation between the logarithm of the initial bacterial DNA template copy number (x-axis) and the corresponding threshold cycle (Ct) values obtained from quantitative PCR (y-axis).





**Figure. S10** **Correlation between bacterial genera and differential metabolites.** Spearman’s correlation heatmap showing the correlation analysis between five bacterial genera and the top 50 differential metabolites. Different colors indicate correlation level; *p < 0.05; **p < 0.01, ***p < 0.001.
